# Cell Cycle-Dependent Dynamics of the Golgi-Centrosome Association in Motile Cells

**DOI:** 10.3390/cells9051069

**Published:** 2020-04-25

**Authors:** Keyada Frye, Fioranna Renda, Maria Fomicheva, Xiaodong Zhu, Lisa Gong, Alexey Khodjakov, Irina Kaverina

**Affiliations:** 1 Department of Cell and Developmental Biology, Vanderbilt University, Nashville, TN 37240, USA; 2 Wadsworth Center, New York State Department of Health, Albany, NY 12208, USA

**Keywords:** interphase, prophase, microtubules, centrosome separation, centrinone, cell motility, Golgi complex

## Abstract

Here, we characterize spatial distribution of the Golgi complex in human cells. In contrast to the prevailing view that the Golgi compactly surrounds the centrosome throughout interphase, we observe characteristic differences in the morphology of Golgi ribbons and their association with the centrosome during various periods of the cell cycle. The compact Golgi complex is typical in G1; during S-phase, Golgi ribbons lose their association with the centrosome and extend along the nuclear envelope to largely encircle the nucleus in G2. Interestingly, pre-mitotic separation of duplicated centrosomes always occurs after dissociation from the Golgi. Shortly before the nuclear envelope breakdown, scattered Golgi ribbons reassociate with the separated centrosomes restoring two compact Golgi complexes. Transitions between the compact and distributed Golgi morphologies are microtubule-dependent. However, they occur even in the absence of centrosomes, which implies that Golgi reorganization is not driven by the centrosomal microtubule asters. Cells with different Golgi morphology exhibit distinct differences in the directional persistence and velocity of migration. These data suggest that changes in the radial distribution of the Golgi around the nucleus define the extent of cell polarization and regulate cell motility in a cell cycle-dependent manner.

## 1. Introduction

The Golgi complex serves as a major hub for intracellular trafficking, allowing for the proper processing of newly synthesized proteins, their sorting, and transport toward proper destinations. The complex comprises polarized stacks of flattened cisternae, which receive proteins translated within the endoplasmic reticulum (ER) at the cis face. After traversing through the Golgi stack and acquiring proper modifications, processed proteins are sorted into post-Golgi carrier vesicles that are primarily targeted for transportation toward the plasma membrane. Due to its vital role in protein trafficking, organization of the Golgi has been studied extensively and the consensus is that the localization and morphology of the Golgi are governed by the cytoskeleton. This governance allows for highly dynamic changes in the organization of Golgi that are paramount for the proper function of this organelle.

In higher eukaryotes, Golgi stacks are linked via tubular connections into a Golgi ribbon whose integrity is important for its role as the central protein processing and sorting station. The integral Golgi complex exists during interphase, but must fragment as the cell enters mitosis [1,2,3]. Fragmentation involves a breakdown of the Golgi ribbon into individual stacks [4], unstacking and vesiculation of Golgi cisternae [5], and cycling of Golgi components through the ER [6,7]. The fragments accumulate near spindle poles organized by the centrosomes, which ensures proper partitioning of Golgi fragments into the daughter cells [8].

Upon mitotic exit, multiple stacks reemerge and rapidly reassemble into the Golgi ribbons in daughter cells [9]. This reassembly is facilitated by transport of ER-to-Golgi carriers and Golgi stacks toward the minus ends of microtubules (MTs) [10,11], driven by the minus end directed molecular motors dynein [12,13,14] and KIFC3 [15]. Transport of Golgi elements relies on two subpopulations of MTs: Golgi-derived MTs are engaged in the transport of Golgi stacks towards each other and are necessary for the ribbon integrity [16,17]**,** while the radially organized MTs produced by the centrosomes collect Golgi membranes to a common complex in the vicinity of the centrosome. Because the centrosome is the most efficient cellular MT-organizing center (MTOC), MT minus end concentration is the highest at the centrosome. As a result, the assembled Golgi ribbon is found closely associated with the centrosome. Additional direct molecular links between the Golgi and centrosomes also support association of those organelles [18,19].

In post-mitotic cells, the centrosomes define the location of the Golgi via these mechanisms. Golgi positioning plays a critical role in cell polarity [20,21], which is particularly important in polarized cells that export post-Golgi proteins in a single defined direction. Such cells position the Golgi near the destination point of secretion. For example, the Golgi is proximal to the apical surface in polarized epithelia that secrete apically [22]. In directionally migrating mesenchymal cells, Golgi resides between the nucleus and the protruding edge, supporting the front-directed Golgi-derived MT array and the transport of the building material to the protruding edge [16,23,24]. The centrosome is thought to drive proper positioning of the Golgi in this context [25].

Although the morphological and functional partnerships between the Golgi and the centrosome appear to be well established, noteworthy is that Golgi assembly and its localization have been primarily assessed in early G1, soon after the cell exited mitosis or in quiescent differentiated cells that have exited from the cell cycle into G0. Morphology and behavior of the Golgi as well as its connections with the centrosome throughout interphase in actively proliferating cells have not been detailed. In this study, we follow the dynamics of Golgi and centrosome positioning throughout the cell cycle in highly motile and rapidly growing RPE1 cells. By combining 3D live-cell time-lapse imaging and quantitative fixed population analyses, we characterize dramatic cell cycle-dependent changes in the localization and morphology of the Golgi ribbon as well as the association of the Golgi and centrosome. We also demonstrate that these dynamic Golgi rearrangements strongly influence the efficiency of polarized cell migration during various phases of the cell cycle.

## 2. Materials and Methods

### 2.1. Cell Culture

Immortalized human retinal pigment epithelial cells hTert-RPE1 cells (CRL-4000, ATCC) were maintained in DMEM/F12 with 10% fetal bovine serum, 100 µM penicillin, and 0.1 mg/mL streptomycin at 37 °C in 5% CO_2_. In all live-cell experiments, cells were maintained on the microscope stage at 37 °C in 5% CO_2_. Cells were plated on glass coverslips or glass bottom dishes (MatTek Corporation, No. 1.5, 14 mm circular) coated with 5 μg/mL fibronectin (EMD Millipore, Burlington, MA, USA) 24 h prior to experiments.

### 2.2. Treatments

For MT depolymerization during live-cell imaging, cells were first treated on ice for 45 min, then the media was replaced with warm media containing 8.3 µM nocodazole (Sigma-Aldrich, St. Louis, MO, USA, M1404). Nocodazole was prepared at 16.6 mM in DMSO. For the centrosome depletion experiments, Centrinone B (Tocris, Minneapolis, MN, USA) was prepared at 1.1 mM in DMSO. Cells were treated with 500 nM for 72 h before live-cell imaging or fixation.

### 2.3. S-Phase Determination

Bromodeoxyuridine (Fisher Scientific, Waltham, MA, USA) was prepared as a stock solution of 10 mM in PBS. Cells were treated with 10 µM BrdU for 5 min before fixation (see below for details). Cells were then treated with 1.5 N HCl for 40 min required for antigen retrieval. A routine antibody staining protocol was utilized after a 15 min PBS wash. Additionally, EdU (ABP Biosciences, Beltsville, MD, USA) was also used to detect S-phase cells. Cells were treated for 5 min with 10 µM EdU before fixation (see below for details). Manufacturer protocol was followed as directed for EdU detection using iClick reaction technology.

### 2.4. Expression Constructs

One of two RPE1 stable cell lines were used for most experiments. (1) Centrin1-GFP was introduced into RPE1 cells by lentiviral incorporation of the construct [26]. (2) Centrin-1-GFP RPE1 cells were then transfected with RFP-TGN (gifted from Enrique Rodriguez-Boulan), [27] via Amaxa (Lonza, Program I-013), then selected with G418 for at least two weeks before cell sorting. ES-FUCCI (fluorescent ubiquitination-based cell cycle indicator, gifted from Pierre Neveu, Addgene plasmid # 62451), mCherry-NLS (gifted from Dylan Burnette), and membrane-anchored GFP-gtn (gifted from Adam Lindsted [28]) were transfected into RPE1 cells using Amaxa or Fugene (Promega, Madison, WI), according to the manufacturer’s protocols.

### 2.5. Immunostaining

For Golgi identification, mouse monoclonal antibody against GM130 (BD Transduction Laboratories, San Jose, CA, USA) or rabbit polyclonal antibody against giantin (Abcam, Cambridge, MA, USA) was used. MTs were stained with anti-α-tubulin rabbit polyclonal antibody (Abcam, Cambridge, MA, USA). S-phase cells were detected by α-mouse BrDU (G3G4 (anti-BrdU)) was deposited to the DSHB by Kaufman, S.J. (DSHB Hybridoma Product G3G4 (anti-BrdU) or iClick EdU Andy Fluor 555 Imaging kit (ABP Biosciences, Beltsville, MD, USA)). For MT and Golgi staining, cells were fixed (15 min at room temperature) in 4% paraformaldehyde, 0.025% glutaraldehyde, and 0.3% Triton in a cytoskeleton buffer (10 mM 2-(*N*-morpholino)ethanesulfonic acid, 150 mM NaCl, 5 mM ethylene glycol tetra-acetic acid, 5 mM glucose, and 5 mM MgCl2, pH 6.1). Alexa 647-conjugated highly cross-absorbed goat anti-rabbit immunoglobulin G (IgG) antibodies and Alexa 568-conjugated goat anti-mouse IgG antibodies (Molecular Probes, Invitrogen, Eugene, OR, USA) were used as secondary antibodies. Coverslips were mounted in Vectashield Mounting Medium (Vector Labs, Burlingame, CA, USA). DNA was detected using either DAPI (Fisher Scientific, Waltham, MA, USA) or Hoechst 33457 (Fisher Scientific, Waltham, MA, USA).

### 2.6. Image Acquisition of Live and Fixed Samples

**Figure 1:**
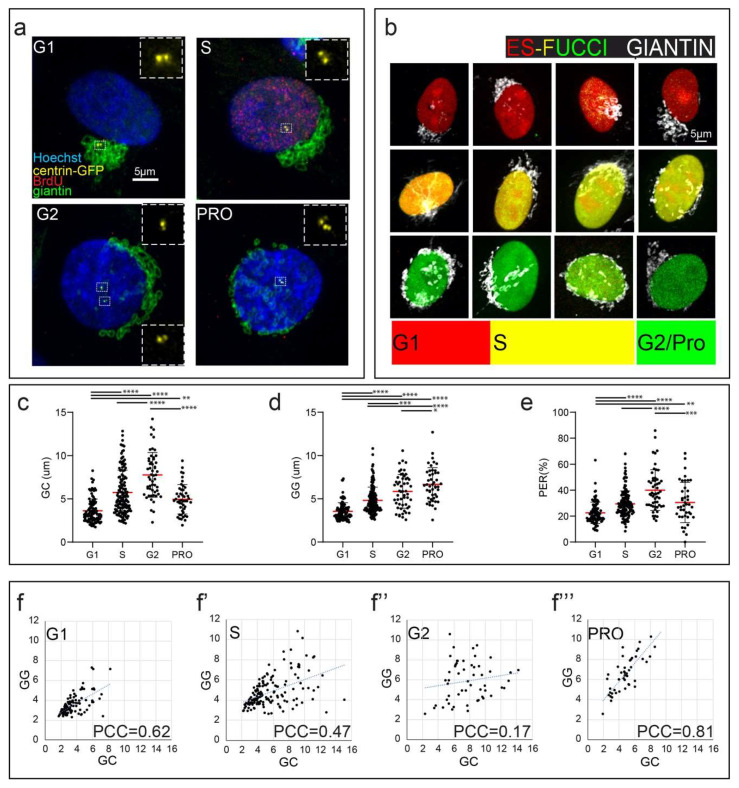
Golgi configuration and centrosome association are cell cycle-dependent. (**a**) Representative images of Golgi and centrosome positioning in specific cell cycle phases (G1, S, G2, prophase (PRO). Immunostaining: Hoechst (blue), centrin1-GFP (yellow), BrdU (red), giantin (green). Boxed regions (centrosomes) are enlarged in insets. Maximum intensity projections of laser scanning confocal stacks. Scale 5 µm. (**b**) Representative images of Golgi (immunostained, giantin, white) throughout the cell cycle as highlighted by ES-FUCCI cell cycle indicator. FUCCI color code: G1, red; S, yellow, G2 and prophase, green). Maximum intensity projections of spinning disk confocal stacks. Scale 5 µm. (**c**–**e**) Quantification of Golgi and centrosome distribution indices in the fixed cell population based on data as in (**a**). *n* = 343 cells total, six experiments. Error bars, SD. Mean, red line. One-way ANOVA with Tukey’s multiple comparison test. **** *p* < 0.0001; *** *p* < 0.001; ** *p* < 0.01; * *p* < 0.05. (**c**) GC (mean Golgi-centrosome distance in 3D space). (**d**) GG (mean Golgi scattering index in 3D space). (**e**) PER (percent of equatorial Golgi redistribution). (**f–f’’’**) Scatter plots of GG vs GC correlation in cell cycle substages. Pearson correlation coefficients demonstrate positive correlation for GG vs. GC in cell stages G1 (**f**) and prophase (**f’’’**).

For images in Figure 1, 3D laser-scanning confocal imaging of fixed cells was performed using Nikon A1r based on a Ti-E inverted microscope with SR Apo TIRF 100× NA1.49 oil lens run by NIS Elements C software (Nikon, Tokyo, Japan).

**Figure 2:**
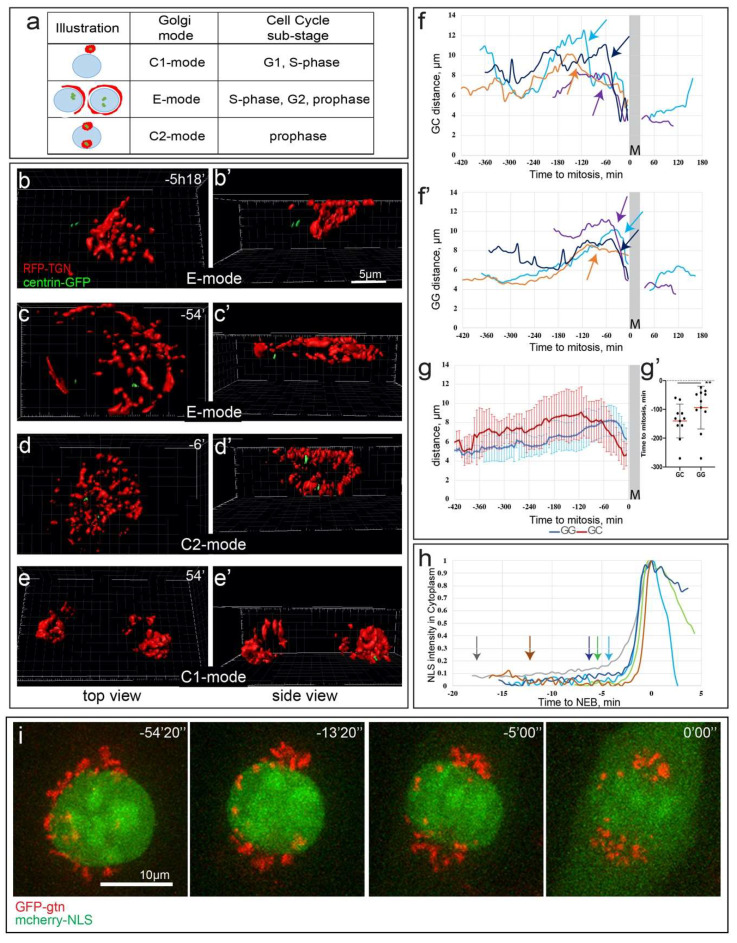
Dynamics and timing of Golgi configuration modes in living cells. (**a**) Model of Golgi mode progression aligned with cell cycle progression. (**b**–**e’**) Frames from a time-lapse imaging sequence of RPE1 cells stably expressing Golgi (RFP-TGN, red) and centrosome markers (centrin1-GFP, green). Corresponding Golgi configuration modes: C1-E transition (**b**,**b’**), E (**c**,**c’**), C2 (**d**,**d’**), C1 (**e**,**e’**). 3D reconstructions of spinning disk confocal stacks (top-down views (**b**–**e**) and side views (**b’**–**e’**)). Time, hours, minutes. (**f**) GC and (**f’**) GG distance profiles for 4 representative cells over time. Time point “0” indicates the beginning of cell division. (**g**) Averaged GC (red) and GG (blue) profiles over time. Sequences aligned by the beginning of cell division (time point “0”). *n* = 9. Error bars, SD. Note the declines of the curves prior to cell division. (**g’**) Time when GC (left) and GG (right) reach maxima before beginning to decline prior to cell division. Data for individual cells analyzed in (**g**). Note that GC peak is significantly earlier than GG. *n* = 9. Red line, mean. Error bars, SD. Student’s *t*-test, ** *p* < 0.01. (**h**) Intensity profiles for NLS signal in the cytoplasm of individual cells prior to and during nuclear envelope breakdown. Based of imaging as in (**i**). C2 mode onset times are indicated by arrows. *n* = 5. (**i**) Frames from a time-lapse imaging sequence of RPE1 cells transfected with GFP-gtn (pseudo-colored red) and mCherry-NLS (pseudo-colored green). Note Golgi compaction at –13′20′′ and –5′00′′ and NLS leakage into the cytoplasm at 0′00′′. Scale bar, 10 µm. Time, minutes, seconds. Images are maximum intensity projection of entire spinning disk confocal stacks.

For Figure 2b–e’ and other 2-color 3D live-cell imaging, we used a spinning disk Yokogawa CSU-X1 confocal based on a Nikon Eclipse Ti-E inverted microscope with an SR Apo TIRF 100× A1.49 oil lens and either Andor DU-897 X-11240 or Photometrics Prime 95B cameras. Z-stacks with planes separated by 0.4 um over the whole cell volume were captured every 6 min.

For Figure 2i, the same spinning disk microscope setup was used with frames captured every 20 s.

**Figure 3:**
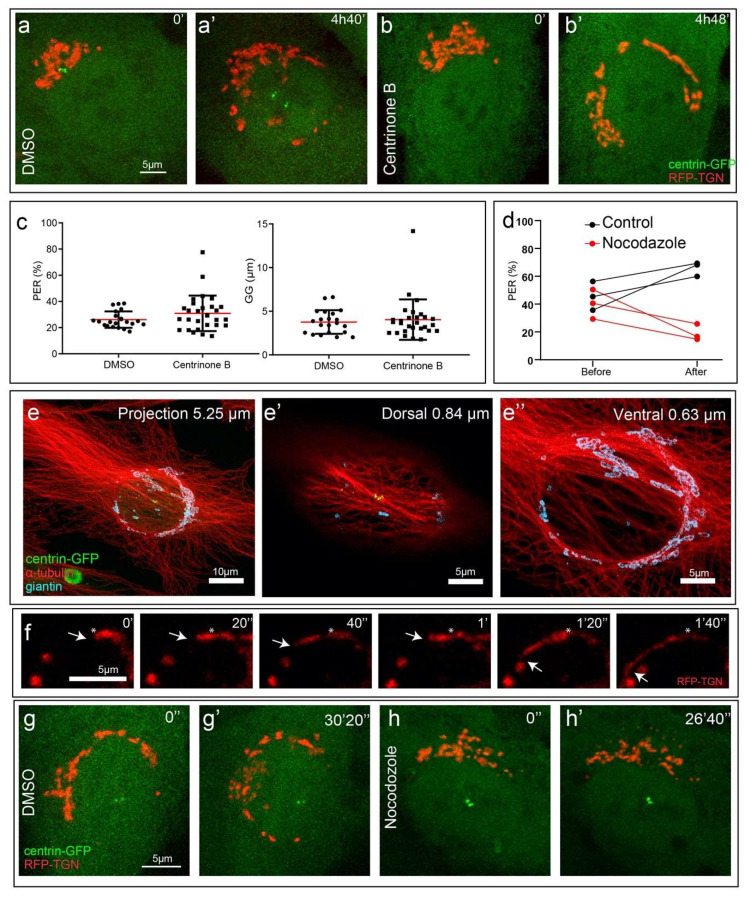
Golgi stretching around the nucleus is centrosome-independent and microtubule-dependent. (**a**–**b’**) Frames from a time-lapse imaging sequence of RPE1 cells stably expressing Golgi (RFP-TGN, red) and centrosome markers (centrin1-GFP, green). E mode progression over 5 h in (**a**) control cell or (**b**) cell pretreated by Centrinone B for 72 h. Scale 5 µm. Time, hours, minutes. (**c**) Quantification of PER and GG in fixed S-phase cells (BRDu-positive) pretreated with DMSO or Centrinone B for 72 h. Student *t*-test showed no significant differences between DMSO and Centrinone B-treated cells for either PER or GG. *n* = 49. Error bars, SD. Red line, mean. (**d**) Change of Golgi extension during 30 min live-cell imaging of cells with and without MTs. Cells pretreated on ice for 45 min were recorded in the presence of DMSO (control) vs. nocodazole. PER index before and after imaging is shown. *n* = 3. (**e**–**e’’**) Localization of the Golgi (giantin, cyan) and MTs (α-tubulin, red) in E mode, immunostaining, centrin1-GFP (green). (**e**) Cell overview shown as a maximum intensity projection of entire laser scanning confocal stack (5.25 µm-thick). Scale 10 µm. (**e’**,**e”**) Maximum intensity projections of dorsal (0.84 µm-thick) and ventral (0.63 µm-thick) sub-stacks of the central cell area. Scale 5 µm. (**f**) Progressive movement (white arrows) of dynamic Golgi tubule (TGN, red) along the nuclear equator shown in 20 s intervals. Asterisk denotes starting position of Golgi membrane tubule movement. Scale 5 µm. Time, minutes, seconds. (**g**) Live cell visualization of E mode progression in cells pretreated on ice for 45 min and recorded in the presence of DMSO (**g**) or nocodazole (**h**, as quantified in **d**). Golgi (RFP-TGN, red). Centrosome (centrin1-GFP, green). Scale 5 µm. Time, seconds. Images in (**a**,**b**,**f**,**g**,**h**) are maximum intensity projection of entire spinning disk confocal stacks.

For Figure 3a,b, and Figure 3g–h’ the same spinning disk microscope setup was used with frames captured every 6 min, and for Figure 3f every 20 s. For Figure 3e–e”, a Leica TCS SP5 microscope with an HCX PL APO 100× oil lens, NA 1.47.

**Figure 4:**
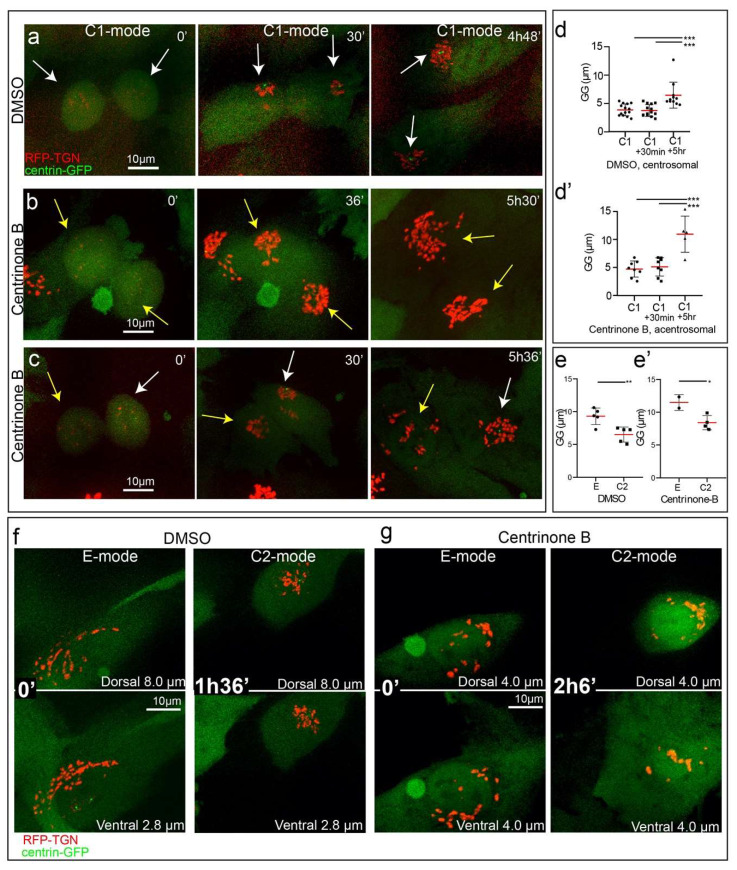
Golgi compaction is centrosome-independent. (**a**–**c**) Frames from time-lapse imaging sequences of dividing RPE1 cells stably expressing Golgi (RFP-TGN, red) and centrosome markers (centrin1-GFP, green), C1 mode progression within a 5 h period in control cell (**a**) or cells pretreated by Centrinone B for 72 h and containing no centrosomes (**b**) or one centrosome (**c**). Daughter cells with centrosomes, white arrows. Daughter cells without centrosome, yellow arrows. Note compact Golgi configuration in all daughter cells after the mitotic exit, and early E mode in acentrosomal cell at 5 h. Scale 10 µm. Time, hours, minutes. (**d**,**d’**) GG distance determined for control (**d**) and acentrosomal (**d’**) cells at 6–12 min after mitotic exit is referred to as “C1”, 30 min and 5 h, referred to as “C1+30min,” and “C1+5h”, respectively. Based on data as in (**a**–**c**). GG is low after mitotic exit regardless of the centrosome presence. At a later time point (5 h), GG in acentrosomal cells is significantly higher than at the earlier time points (Student *t*-test, *** *p* < 0.001). It is also significantly higher than GG at 5 h time point in DMSO (Student *t*-test, (*p* < 0.01). *n* = 11). (**e,e’**) GG distance determined for E mode to C2 mode transition in RPE1 cells stably expressing Golgi and centrosomes. Control (DMSO, **e**) does not significantly differ from acentrosomal cells (Centrinone B, **e’**). Student *t*-test showed significant differences (** *p* < 0.01) between E mode and C2 mode after DMSO treatment and significant difference (* *p* < 0.05) between E mode and C2 mode in Centrinone B treated cells. *n* = 3–4. Error bars, SD. Red line, mean. (**f**,**g**) Frames from time-lapse imaging sequences of dividing RPE1 cells stably expressing Golgi (RFP-TGN, red) and centrosome markers (centrin1-GFP, green), E mode to C2 mode transition within a 2 h period in control cell (**f**) or cells pretreated by Centrinone B for 72 h and containing no centrosomes (**g**). Images are maximum intensity projection of dorsal or ventral part of spinning disk confocal stacks. Size of stack is indicated in each image. Scale, 10 µm. Time, hours, minutes.

For all images in Figure 4 the same spinning disk microscope setup was used with frames captured every 6 min.

For Figure 4a, centriole behavior was visualized in multi-mode (DIC and wide-field epifluorescence) 3D time-lapse recordings on a Nikon TE20002E microscope equipped with a 100× 1.45NA PlanApo lens. Seventeen z-planes separated by 0.5 um were captured every 5 min for both modalities. Images were recorded on an Andor iXon DU897 camera at 107 nm/pixel magnification. The microscope was controlled by IPLab software (Scanalytics, Milwaukee, WI, USA). 3D movements of mother centrioles were tracked using Imaris software (Bitplane, Concord, MA, USA).

**Figure 5:**
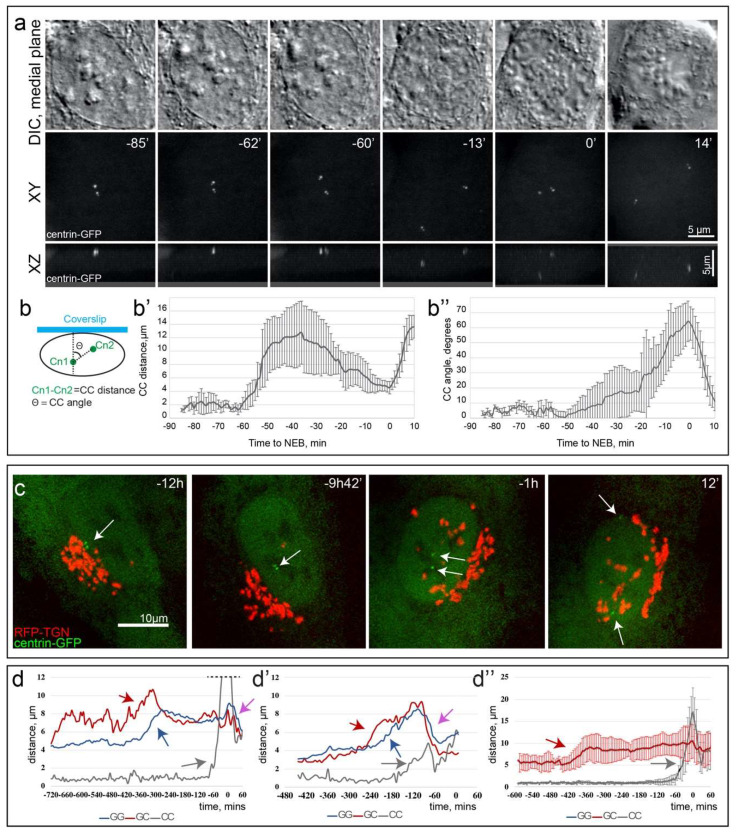
Centrosome separation occurs in E mode, after dissociation from the Golgi. (**a**) Frames from multi-mode DIC (upper row) and wide-field epifluorescence 3D time-lapse recordings. GFP-centrin is shown as maximum intensity projections (middle row, XY view; lower row, XZ view). Arrows indicate centrosomes. Scale 5 µm. Time point “0” indicates NEB. Time, minutes. (**b**–**b”**) Quantification of centrosome positioning in data like in (**a**). (**b**) Schematic of centrosome-centrosome (CC) distance and angle quantification. (**b’**) Dynamics of CC distance over time. (**b’’**) Dynamics of CC angle over time. *n* = 13. Error bars, SD. (**c**) Frames from a time-lapse imaging sequence of RPE1 cells stably expressing Golgi (RFP-TGN, red) and centrosome markers (centrin1-GFP, green) during E mode progression. White arrows indicate centrosome position. Scale 10 µm. Time points correspond to values approaching “0” which indicates the maximal CC. Time, hours, minutes. Maximum intensity projection of entire spinning disk confocal stacks. (**d**,**d’**) GG (blue line), GC (red line), and CC (grey line) distance profile for representative cells. Grey arrows indicate the CC distance increases after increase of GG (blue arrows) and GC (red arrows), but prior to GG/GC decreases (purple arrows) indicating C2 mode. Top of graph in 5d was cut to highlight changes in GG/GC/CC distances. Time point “0” indicates the maximal CC. Time, minutes. (**d”**) Averaged GC (red) and CC (gray) profiles over time. Sequences aligned by the maximum CC (time point “0”). Note that GC (red arrow) increases significantly earlier than CC (grey arrow). *n* = 11. Error bars, SD. Time, minutes.

**Figure 6:**
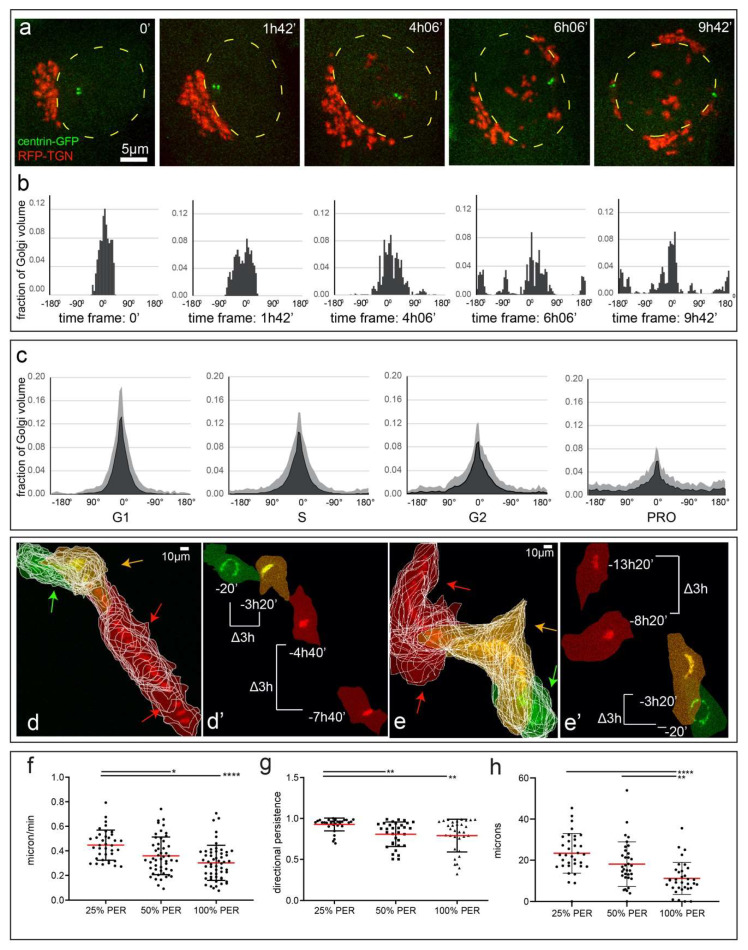
C1 Golgi compaction correlates with high cell migration speeds. (**a**) Frames from time-lapse imaging sequences of an RPE1 cell stably expressing Golgi (RFP-TGN, red) and centrosome markers (centrin1-GFP, green) during E mode progression. Yellow dotted lines indicate nucleus outline. Maximum intensity projections of laser scanning confocal stacks. Scale 5 µm. (**b**) Histograms of radial Golgi distribution in the cell shown in (**a**), corresponding to each timepoint above. Fraction of Golgi volume in 5-degree radial sectors are plotted. Angle 0 indicates the bin with the largest number of Golgi voxels. (**c**) Mean histograms of radial Golgi distribution per cell cycle stage for entire fixed cell population (as used in Figure 1b–d). SD is shown in light gray. (**d**–**e’**) Overlaid frames from time-lapse epifluorescence imaging sequences of RPE1 cells stably expressing Golgi marker (RFP-TGN). Images are pseudo-color-coded according to Golgi configuration modes: compact Golgi (PER < 25%, red), early E mode (PER = 25–50%, yellow), and advanced E mode (PER > 50%, green). (**d**,**e**) Frames with 10 min intervals are overlaid. Cell outlines are shown in white. Note large displacements between cells with compact Golgi (red arrows), and smaller displacements between cells with early (yellow arrows) and advanced (green arrows) E mode. (**d’**,**e’**) Selected frames with 3-h intervals between cells in C1 (red) and E mode (yellow, green). Note that displacement (Δ3h) for C1 mode is larger than for E mode. (**f**–**h**) Cell migration parameters according to the Golgi configuration (PER). (**f**) Speed measured in microns per minute. (**g**) Directional persistence (0–1; 0 indicating random cell trajectory, 1 indicating straight trajectory). (**h**) Displacement measured in microns over one hour. One-way ANOVA with Tukey’s multiple comparison was used to determine differences (* *p* < 0.05, ** *p* < 0.01, **** *p* < 0.0001). Scale 10 µm. Time, hours, minutes. Error bars, SD. Red line, mean.

For Figure 5c and Figure 6a, a spinning disk setup described above for Figure 2 was used with frames captured every 6 min.

For Figure 6d,e, cell migration recording was performed as semi-simultaneous wide-field fluorescent and DIC imaging (10 min/frame) at an inverted Nikon TE2000E microscope with a Perfect Focus System using a 20× lens and a Photometrics CoolSnap HQ CCD camera driven by IPLab software (Scanalytics, Milwaukee, WI, USA).

### 2.7. Quantitative Analyses

#### 2.7.1. Centrosome Distance Parameters

Centrosome position was determined using automatic detection of the intensity maximum of the centrioles using Imaris program (Bitplane). Both centrioles were tracked in G1 cells, and mother centrioles in S and G2 cells. For live image sequences, manual tracking implementing the same algorithm was used. Centrosome-to-centrosome distance (CC), 3D, was calculated by measuring the distance of centrosome foci in a single cell over time. Centrosome-Golgi separation is defined as the centrosomes moving greater than 3 µm away from the Golgi mass. Centrosome-centrosome separation is defined as duplicated centrosomes moving greater than 2.5 µm away from each other.

#### 2.7.2. Golgi and Centrosome Positioning Parameters

For Golgi segmentation, the level of background was determined based on intensity histograms of maximum intensity projections, where the background intensities show as a gaussian distribution in the low part of the histogram. The threshold at 5% intensity above the background was thereafter implemented to full 3D image stacks. Importantly, the background was identified individually for each live imaging sequence to accommodate variability of the Golgi marker expression level. For fixed images, the background was identified per experimental repeat to account for variability of immunostaining intensity (Supplementary Materials Figure S1a).

For Golgi to centrosome distance (GC), the distance from each voxel within segmented volume of the Golgi to both centrosomes was determined, and the mean distance to the nearest centrosome was calculated to describe Golgi association with the proximal centrosome. The spread of Golgi (GG) was calculated as the mean of pairwise distances for all voxels within segmented volume of the Golgi. Both parameters were calculated in Matlab (MathWorks, Natick, MA, USA). Percentage of equatorial redistribution (PER), 2D, was determined by manually measuring the nuclear perimeter and the length of Golgi less than 2 µm away from nucleus in a maximum intensity projection of each cell. Calculating the ratio of the two measures produces PER values.

#### 2.7.3. Radial Distribution of Golgi

Radial distribution of Golgi was assessed by the number of voxels within 5-degree segments in 2D polar coordinates with the origin coinciding with the center of the nucleus (Figure S3). Angle 0 was assigned to the center of the bin with the largest number of segmented voxels, i.e., the largest volume of Golgi. Radial histograms were calculated in Matlab (MathWorks, Natick, MA, USA).

#### 2.7.4. Nuclear Envelope Breakdown (NEB)

To determine point of NEB, maximum intensity projections of the mCherry-NLS channel were registered with the ImageJ StackReg plugin, using the translation function. An ROI was drawn in the cytoplasm of each cell and the mean intensity of the selection was measured and plotted overtime. Intensity curves of 8 cells were normalized and aligned based on a value of 1, indicating the time point where the NLS signal was the highest in the cytoplasm. We consider this the point of NEB. Golgi compaction in Golgi mode C2 was determined independently of NLS leakage. The Golgi channel was observed for the changes of the Golgi configuration. The timepoint where there was detection of Golgi particles moving toward spindle poles is designated as the start of C2. The start of C2 and the peak of NLS intensity of several cells were then plotted on one graph to determine any correlation of the timing of the two events.

#### 2.7.5. Cell Migration Speed, Displacement, and Directional Persistence

RPE1 cells expressing RFP-TGN were imaged using spinning disk microscopy. Both the Golgi fluorescence and DIC images were recorded. DIC was used to track the cell trajectory every 10 min using the MTrackJ plugin in ImageJ. The center of the nucleus was used as a reference point for tracking. All movies used for analysis were 60 min long. To determine changes in cell migration parameters (speed, displacement, directional persistence) output from this tracking macro was utilized. Cells were grouped into three groups, based on each cell’s PER. “<25% PER” includes cells that were newly divided within the time lapse of the movie, with PER less than 25%. “25–50% PER” includes cells with PER between 25% and 50%. “>50% PER” included cells with PER greater than 50% that were also shown to enter mitosis within the time lapse of the movie. For each cell track, displacement was measured as the distance from start (D2S). Directional persistence was calculated as the maximum distance from the start of the track (D2S) divided by the length of the track (Len). Values approaching 1 indicate a unidirectional track.

### 2.8. Cell Cycle Analysis

BrdU (EdU) was added to culture medium of a non-synchronized population of centrin1-GFP-expressing RPE1 cells. After a five-minute BrdU (EdU) incorporation, cells were fixed and processed for immunostaining to highlight Golgi, chromatin (Hoechst) and BrdU (EdU) incorporation. BrdU (EdU)-positive cells were considered S-phase cells. Cells with condensed chromosomes and intact nucleus were considered prophase. Cells with condensed chromosomes and no intact nucleus were considered in post-NEB mitosis. Cells with two centrioles were considered G1. Cells with four centrioles and no BrdU (EdU) incorporation were considered G2. Cells where centrioles number could not be identified and no BrdU (EdU) incorporation were excluded from the analysis.

### 2.9. Statistics

All quantitative data were collected from experiments performed in triplicate or more, unless mentioned in the figure legends. Data sets were analyzed using a one-way ANOVA *F*-test, Tukey’s multiple comparison test, and/or Student’s *t*-test, as described in the figure legends.

### 2.10. Image Processing

Image analysis was performed using ImageJ, Matlab, and Imaris. 3D image reconstruction was made using Imaris. For all fluorescence images presented here, adjustments were made to brightness, contrast, and gamma settings to make small structures visible.

## 3. Results

### 3.1. Morphology of the Golgi Complex and Its Association with the Centrosome Varies Depending on the Cell Cycle Stage

Analysis of the Golgi morphology and its association with the centrosome in fixed cells randomly selected from asynchronous populations of human euploid RPE1 line suggests prominent cell cycle specific differences in the organization of this organelle. To identify cells during various phases of the cell cycle, we briefly expose RPE1 cells that constitutively express a GFP fusion of centriolar protein Centrin-1 [26] to BrdU. In this approach, nuclei of cells in the S-phase become labeled due to incorporation of BrdU while G1 and G2 cells remain unlabeled (Figure 1a). The latter stages are discriminated by the organization of the centrosome as G1 cells contain two individual centrioles while G2 cells display two centriolar doublets (diplosomes) (Figure 1a) [26,29]. Prophase was identified as the period when the condensed chromosomes within the intact nucleus can be morphologically detected (Figure 1a). In an alternative approach, we employ Fluorescent Ubiquitination-based Cell Cycle Indicator (FUCCI [30]) (Figure 1b). Both approaches yield similar results. Consistent with previous reports [16,31], the Golgi appears compact and closely associated with the centrosome in G1 cells. In contrast, Golgi-centrosome association is not apparent at later stages of the cell cycle (Figure 1a).

To quantify spatial proximity of the centrosome to the Golgi, we employ the mean of distances between the centriole and each of the voxels within segmented volumes of the Golgi (Golgi-Centrosome distance, GC, Figure S1a). GC values are low in G1 phase but higher and more variable among S-phase cells (Figure 1c). These differences indicate that the Golgi spreads away from the centrosome as the cell progresses through S-phase. Morphologically observed increases in GC values correspond to Golgi extension along the nuclear envelope (Figure 1a, ‘S’ and ‘G2′), which we describe as the ‘equatorial’ or ‘E mode’ configuration. In cells with equatorial Golgi, the centrosome tends to separate from the Golgi ribbons and assume position closer to the center of the nucleus either at its ventral or dorsal surface (Figure 1a, ‘S’). In G2, GC values are consistently high, while the centrosome remains detached from the equatorially distributed Golgi (Figure 1a, ‘G2′). In prophase cells, GC values decrease as the Golgi appear to reassociate with the centrosomes that continue to reside beneath or above the nucleus near its center (Figure 1c).

We use two parameters to numerically assess changes in Golgi morphology: Golgi-to-Golgi index (GG) and percent of equatorial redistribution (PER). The former reflects the extent of Golgi fragmentation and scattering and is calculated as the mean of pairwise distances among all voxels within the segmented volume of the Golgi (Figure S1a). The latter is proportional to the extension of the Golgi around the nuclear equator and is measured as the percent of the nuclear perimeter that is in close proximity with the Golgi (Figure S1a). We observe a strong correlation between the values of GG and PER during all cell cycle phases (Figure S1b), allowing us to use either type of analysis as needed.

We find that in G1, both metrics are low (Figure 1d,e), consistent with the observed compact morphology of the Golgi. Henceforth, we will reference this quantifiable morphology as Compact configuration mode-1 (C1 mode). Interestingly, we find a strong direct correlation between the metrics describing Golgi-centrosome association (GC) and Golgi scattering (GG) during G1, supporting the idea that collecting the Golgi around the centrosome is an important factor in Golgi compaction in G1 cells (Figure 1f).

In S-phase and G2, GG and PER values progressively increase, as many cells reach the extended Golgi configuration (E mode) (Figure 1d,e). However, variance of both metrics is high in S-phase consistent, with the visually observed variability of Golgi morphology and centrosome configurations (Figure S2). It is not uncommon to observe S-phase cells with centrosomes spatially separated from compact Golgi complexes (Figure S2c). Conversely, on occasion the Golgi is found in E mode while the centrosome resides adjacent to Golgi ribbons (Figure S2b). Consistent with these observations, only a weak correlation between GC and GG is observed for S-phase cells (Figure 1f’). This variability suggests that centrosome detachment and early stages of Golgi equatorial spreading are roughly coincidental but not necessarily interdependent. In G2, when the Golgi is in E mode, the correlation between GG and GC is not detected (Figure 1f”), indicating that the Golgi and the centrosome positioning become completely independent from each other.

In prophase cells, as the Golgi undergoes pre-mitotic fragmentation, the mean value of GG remains similar to that observed in G2 cells (Figure 1d). In contrast, the mean value of PER decreases (Figure 1e). This decrease reflects a change in the spatial distribution of Golgi fragments, which becomes pronouncedly three-dimensional. While in S and G2 cells the Golgi spreads around the nucleus primarily in the medial plane, during prophase a number of Golgi fragments reside beneath and/or above the nucleus on its ventral and dorsal surface (Figure S2e–h). Interestingly, GG and GC values correlate strongly in prophase cells (Figure 1f”’), suggesting that the activity that collects the Golgi at the centrosomes is restored akin to the G1 configuration. Indeed, in a subpopulation of prophase cells the Golgi, while highly fragmented, accumulates in the proximity of the separated centrosomes (Figure S2h and low GC in Figure 1c). This configuration will be referred to as Compact-2, or C2 mode.

Overall, fixed-cell analyses allow us to postulate the existence of three Golgi configuration modes (Figure 2a), indicating that centrosome-associated compact Golgi, thought to be typical for all interphase cells [10,32], is rarely observed during S and G2 phases.

### 3.2. Golgi Configuration Dynamics Is Governed by the Cell Cycle Progression

To characterize dynamics of Golgi-centrosome relationships and transitions between the compact and spread configurations of the Golgi, we employ 3D live-cell time-lapse imaging of cells stably co-expressing centrin1-GFP (centrosome marker) and RFP-TGN (Golgi marker). Initial spreading of the Golgi along the nuclear equator roughly coincides with the centrosome detachment from the Golgi (Figure 2b,b’, Video S1) and this spreading steadily advances until full equatorial extension (E mode) is achieved (Figure 2c,c’, Video S1). The raise in GC values precedes that in GG, suggesting that Golgi decompaction follows its dissociation from the centrosome (Figure 2f,f’,g). Consistent with our fixed-cell analyses (Figure 1b, Figure S2e–h), shortly before cell division the Golgi is seen accumulating around the separated centrosomes (C2 mode; Figure 2d,d’, Video S1) and the GC value decreases (Figure 2f, arrows). Live-cell recordings also reveal a transient change in the compactness of Golgi prior to cell division (Figure 2f’, arrows). During this change, GG values decrease quite rapidly, which makes detection of this transient pre-mitotic compaction difficult in fixed-cell population analyses. Importantly, the GG decrease occurs with a slight delay after the decrease in GC (Figure 2g,g’) indicating that the movement of the Golgi toward the centrosome precedes compaction of the Golgi.

To precisely time C2 mode with respect to the NEB (onset of cell division), we correlate dynamics of the Golgi reorganization with redistribution of mCherry-NLS from the nucleus into the cytoplasm (Figure 2h,i, Video S2). In seven of eight recorded cells, the directional movement of Golgi fragments toward the centrosomes is detected prior to the increase in nuclear envelope permeability (Figure 2h,i), suggesting that transition to C2 mode is driven by prophase-associated mechanisms. Upon completion of cell division, the Golgi acquires compact morphology around the centrosome as manifested in the low values of GG and CG characteristic of G1 phase of the cell cycle (C1 mode, Figure 2e,e’,f,f’, Video S1).

Overall, live-cell microscopy illustrates the dynamic nature of the three Golgi modes, and reveals that transition from C1 to E mode occurs during S-phase, E to C2 mode transition takes place during late prophase, and the compact C1 mode Golgi is assembled coincidentally with mitotic exit.

### 3.3. Transition from Compact to Equatorial Golgi Configuration Requires Microtubules but Not Centrosomes

Live-cell recordings indicate that the centrosomes detach from the Golgi during the C1 to E mode transition. To evaluate whether the centrosome plays an active role in this process, we utilize a PLK4 inhibitor Centrinone B known to prevent centriole duplication [33]. Due to dilution of non-duplicating centrioles in the consecutive cell cycles, many cells in the population bear a single centriole or lack this organelle completely after 72 h of Centrinone treatment [33]. We find that acentrosomal cells undergo normal transition to E mode Golgi (Figure 3a,a’, Video S3 vs. Figure 3b,b’, Video S4) as evident from similar values of GG and PER in centrosomal vs. acentrosomal cells (Figure 3c). Thus, the centrosome and by inference MTs organized by this organelle are not required for C1 to E mode Golgi transition.

Lack of dependency on the centrosome-organized astral MT array suggests that E mode Golgi movements rely on non-radial perinuclear MTs that wrap around the nucleus and are closely associated with the extended Golgi (Figure 3e–e’’). Consistent with this notion, rapid directional sliding of Golgi tubules along the nuclear perimeter is often observed in live-cell recordings (Figure 3f, Video S5). Such fast directional movements are typical for active MT-dependent transport [34]. To test whether MTs indeed serve as tracks for E mode Golgi distribution, we utilize a cold treatment that leads to MT depolymerization without Golgi dispersion [35,36], and follow the Golgi dynamics for 30 min at 37 °C in the presence of DMSO (Figure 3g,g’, Video S6) or nocodazole (Figure 3h,h’, Video S7), which prevents MT reassembly. In the absence of MTs, Golgi scattering occurs as expected [35], but directional Golgi movements as well as progression towards E mode are blocked as manifested in lower PER values in three independent image sequences (Figure 3d). This indicates that the C1 to E mode Golgi redistribution, while not centrosome-dependent, is driven by MT-dependent transport.

### 3.4. Golgi Compaction Does Not Require Centrosomes

Our data reveal that compaction of the Golgi occurs twice during a cell cycle: first, upon mitotic exit in G1 and second, during prophase shortly before the NEB. In both cases, the Golgi appears to compact around the centrosome and it is well documented that Golgi stacks are brought together by a MT minus end-dependent transport [37] with both centrosomal and Golgi-derived MTs serving as tracks for Golgi assembly [16,17]. This prompted us to test whether centrosomes play an active role in the transitions to C1 and C2 mode of Golgi.

To test whether the centrosome and centrosomal MT aster are required for proper Golgi compaction, we compare dynamics of transitions to C1 and C2 mode in cells with normal centrosomes (Figure 4a, Video S8) vs. cells lacking centrosomes (Figure 4b, Video S9) vs. cells with a single centriole (Figure 4c, Video S10). Surprisingly, we observe neither a significant difference in Golgi organization (Figure 4a–c, ~30 min time point), nor a significant change in the mean GG value (Figure 4d,d’, C1, C1+30min) in centrosomal vs. acentrosomal cells shortly after mitotic exit. Thus, the centrosome and centrosomal MT asters are not required for Golgi compaction during mitotic exit which suggests that non-centrosomal MTs are sufficient for transport of Golgi fragments toward one another at this stage. However, the Golgi in acentrosomal cells becomes decompacted earlier than in control (compare time points “C1+5h” in Figure 4d,d’), and a premature redistribution of the Golgi to equatorial configuration is observed in a subset of acentrosomal cells (compare centrosomal vs. acentrosomal daughter cells in Figure 4c, 5h36′). This indicates that the compact Golgi is somewhat unstable in the absence of centrosomes consistent with a decrease in Golgi integrity reported previously for acentrosomal cells [17].

Similarly, transition to C2 mode during prophase occurs efficiently in acentrosomal cells (Figure 4e–g). As in the presence of centrosomes (Figure 4f, Video S11), two distinct Golgi clusters form on the dorsal and ventral sides of the nucleus when the centrosomes are absent (Figure 4g, Video S12). Normal extent of pre-mitotic compaction in the absence of centrosomes is evident from similar sharp decreases in GG values in centrosomal vs. acentrosomal cells (Figure 4e,e’). We therefore conclude that the mechanism(s) driving both C1 and C2 modes do not require centrosomes or radial MT arrays formed by these organelles.

### 3.5. Centrosomes Are Dissociated from the Golgi during Pre-Mitotic Centrosome Separation

Thus far, our data demonstrate that the Golgi has a reproducible pattern of association and dissociation from the centrosome within the cell cycle, and that transitions between the compact and spread Golgi morphologies occur in a centrosome-independent manner. This prompted us to investigate whether the Golgi influences behavior of the centrosome.

The two centrioles inherited by each cell during mitosis form a common complex in the majority of RPE1 cells [38] which often resides near the Golgi between the nucleus and the advancing edge of the cell [39]. Consistent with this notion, in fixed-cell analyses we observe short centriole–centriole (CC) mean distances in G1 (0.62 ± 0.27 µm, *n* = 104) and S (0.74 ± 0.51 µm, *n* = 151) phases. Both mean CC distance and variance of the distribution increase significantly in G2 cells (2.48 ± 3.71 µm, *n* = 54) and the mean further increases among prophase cells (5.14 ± 3.24 µm, *n* = 47). These changes suggest that the common complex comprising two mature centrioles breaks down some time in G2 and the released centrioles move away from each other. This behavior is expected as the two centrosomes are consistently found on the opposite sides of the nucleus either on its dorsal and ventral surfaces (~75% of cells [40]), or near the opposite edges near the nuclear equator (~25% of cells, [40]). To detail the pattern of centrosome separation and correlate it with the behavior of Golgi, we followed the behavior of centrioles labeled via constitutive expression of centrin1-GFP (Figure 5a, Video S13). Analysis of 13 cells suggests that the duplicated centrioles (diplosomes) begin to separate ~1 hr prior to NEB. The diplosomes initially move away from each other along the nuclear envelope on the same side of the nucleus. In ten cells, upon reaching the opposite edges of the nucleus, one diplosome reverses its direction and moves back towards its original position, while the second diplosome moves over the edge and towards the center of the nucleus but on its opposite surface (Figure 5a, Video S13). As a result of this complex pattern, CC transiently increases and then decreases as the centrosomes settle near the nuclear center on the ventral and dorsal surfaces. In the remaining three cells, upon the initial separation, the centrosomes remain at the opposite nuclear edges until NEB (our unpublished observations). Germane in these observations is that separation of diplosomes from the common complex occurs late in G2 when, as described above, the Golgi is already dissociated from the centrosome.

These observations are consistent with a concurrent recording of Golgi and centrosome behaviors within the same cell (Figure 5c,d, Videos S14 and S15). In all 27 recorded movie sequences, the centrosomes were released from the common complex and started moving apart (CC distance > 2.5 µm) significantly later than dissociation of the centrosome from the Golgi (295 ± 179 min; min = 42 min; max = 648 min). A sharp increase in CC distance (gray arrows in Figure 5d–d”) consistently occurs after the Golgi-centrosome dissociation (GC increase, red arrows in Figure 5d–d’’). Consistently, at the time of the centrosome separation, Golgi has normally already acquired E mode configuration around the nucleus (high GG levels, blue arrows in Figure 5d,d’). Furthermore, the CC distance increase occurs prior to a sharp decline in GC and GG, indicative of C2 mode Golgi compaction around the centrosome (purple arrow in Figure 5d,d’).

We conclude from these data that centrosome separation occurs within the period between the centrosome-Golgi dissociation and initiation of Golgi compaction. This is also the period when no correlation is observed between the Golgi-centrosome association and Golgi positioning, thus these two organelles are independent of each other. Together with the previous finding that blocking Golgi fragmentation inhibits the separation of mitotic spindle poles [41], our data suggest a possibility that dissociation of the Golgi from the centrosome in E mode allows for the subsequent centrosome separation process.

### 3.6. Compact Golgi Mode Is Associated with Highest Cell Migration Speeds

The highly reproducible pattern of the Golgi morphological reorganization suggests that the process is functionally significant, although the exact role of each configuration is not immediately apparent. We reasoned that one consequence of a highly compact Golgi in a motile cell is that a cell acquires a high degree of polarity due to a narrow radial distribution of the Golgi. This prompted us to assess changes in the radial distribution of Golgi with respect to the nucleus (Figure S3) as a cell progresses through interphase (Figure 6a,b, Video S16). When the Golgi is in C1 mode, radial histograms centered on the center of the nucleus display a single narrow peak expected for highly polarized cells (Figure 6b, time 0). This trend was typical for the overall G1 cell population (Figure 6c). As the cell progresses through the cell cycle, this peak becomes wider as the Golgi gradually spreads equatorially (Figure 6b,c). This indicates a gradual decrease in polarity throughout interphase and prophase.

Because Golgi integrity and positioning are important factors in motile cell polarity [20] and Golgi scattering impairs directional cell migration [16,23,24], we correlate radial distribution of the Golgi with the velocity of cell migration in motile cells over the course of interphase. We observe that cells with compact Golgi move more efficiently and cover longer distances (Figure 6d–e’, Videos S17 and S18) than cells with Golgi positioned more equatorially. Further, we observe a clear correlation between the value of PER that characterizes equatorial spread of the Golgi and the main parameters of migration (Figure 6). Specifically, cells with PER values lower than 25% (typical for cells that exited mitosis recently) display a higher velocity (Figure 6f), higher directional persistence (Figure 6g), and longer displacements (Figure 6h). Thus, Golgi compaction appears to define the extent of motile cell polarization and influence cell motility in a cell cycle-dependent manner.

## 4. Discussion

**Figure 7:**
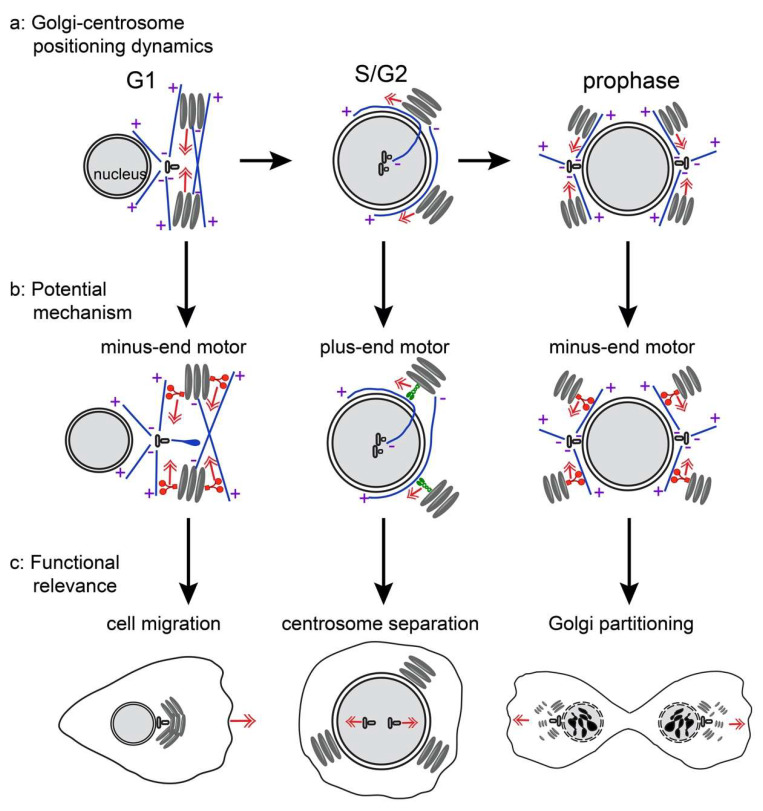
Model of Golgi-centrosome relationships throughout the cell cycle. (**a**) Golgi and centrosome association and positioning in G1 (C1 mode, left), S/G2 (E mode, center), and prophase (C2 mode, right). Both centrosomal and Golgi-derived MTs serve as tracks for Golgi movement. (**b**) Proposed mechanism driving Golgi configuration in the stages according to those depicted in (**a**). Because the centrosomal MT aster is dispensable for the Golgi configuration, it is likely that switches in molecular motor activity are involved. Minus end directed motor activity prevails in compact Golgi modes (**b**, left and right). Plus end directed motor transport along peri-nuclear MTs prevails during E mode (**b**, center). (**c**) Cellular functions that correlate with Golgi configuration modes depicted in (**a**), based on our findings and the literature. Compact Golgi in G1 supports cell polarity and migration (**c**, left). Detachment of the centrosome from the Golgi in E mode allows for the pre-mitotic centrosome separation (**c**, center). Concentration of Golgi fragments at the mitotic poles support efficient Golgi partitioning during cell division (**c**, right).

This study highlights the dynamicity of the Golgi complex’s morphology and its association with the centrosome throughout the cell cycle substages. Several key findings can be taken from this work. First, the Golgi complex transitions between periods of high compactness around the centrosome and perinuclear extension away from the centrosome (Figure 7a). These configuration modes are highly correlative with the cell cycle substages. Compact mode 1 (C1 mode) corresponds to cells newly exited from mitosis, thus G1 cells (Figure 7a, left). The equatorial mode (E mode) is characterized by dissociation of the centrosome from the Golgi and proximity of the Golgi and the nuclear envelope. The degree of Golgi extension around the nucleus increases during S-phase and culminates with the Golgi complex stretched around the perimeter of the nucleus at late G2 or early prophase (Figure 7a, center). Next, there is a quick flip to a second state of high Golgi compactness close to the centrosomes, usually directly prior to the NEB (Figure 7a, right). Our data indicate that there is a regulated switch from the compact to extended Golgi configuration in S-phase and an opposing switch from extended to compact configuration in prophase. It is reasonable to imply that the mechanism driving Golgi compactness remains activated throughout mitosis to keep remaining Golgi elements proximal to the spindle poles during division [8], and driving compaction of emerging Golgi in daughter cells.

In addition to describing the dynamic changes in interphase cells, our data show the contribution of microtubules and centrosomes in cells adopting these described Golgi membrane configurations. In accordance with all prior research, we show that Golgi morphology and rearrangement absolutely rely on MTs and MT-dependent transport (Figure 7b). Because all Golgi modes persist in acentrosomal cells, it is likely that non-centrosomal Golgi-derived MTs are sufficient to support Golgi rearrangements, as we have previously demonstrated for Golgi complex assembly [16,17]. The role of the centrosomes is more complex. Prior research has shown the Golgi complex and the centrosomes are closely associated in interphase [25,42]. This connection is initiated and maintained by the MT motor dynein which positions ER-to-Golgi carriers and Golgi membranes at the concentration of MT minus ends around the centrosome [16,28,31,43]. Additionally, linker proteins like AKAP450 [44] or GM130 [18] were implicated in directly connecting the two organelles. The importance of interphase Golgi-centrosome association for cell polarity, migration, ciliogenesis, and other functions was suggested in a number of studies [44], but challenged by others [18]. Another solid body of studies indicates that fragmented Golgi elements are concentrated around the centrosome in mitosis to support equal partitioning of Golgi membranes into daughter cells [45]. This concept was also challenged by the finding that Golgi can reemerge from the ER upon mitotic exit, thus equal partitioning might not be absolutely necessary [2,43].

Our study is unique because it determines a cell cycle time frame when Golgi-centrosome association does not apply, highlighting the necessity of controlling this bond and indicating that it is indeed an important cellular function, and not a coincidence. Moreover, we find that the centrosome separation prior to mitosis occurs within this time frame (Figure 7c, center). Interestingly, prior studies have found that artificial blocking of Golgi dynamics results in monopolar mitotic spindle assembly (known to arise from a lack of centrosome separation), interpreted as a necessity for Golgi disassembly in mitosis [41]. However, our study shows that the Golgi-centrosome bond is dissolved in interphase and reestablished in prophase following centrosome separation. Combined, prior and our data indicate that it is important to dissociate the Golgi from the centrosome prior to mitosis for efficient centrosome separation and bipolar spindle assembly, while these two organelles reassociate at the mitotic entrance (C2 mode) to assure perfect Golgi partitioning (Figure 7c, right).

On the other hand, we find that Golgi compaction itself, and not only the Golgi-centrosome bond, is a cell cycle-regulated phenomenon, because in acentrosomal cells it occurs at the same stages as in control. These data suggest that the cell cycle switch that initiates Golgi configuration modes likely involves change in molecular motor activity. In particular, a minus end directed motor must prevail during G1 and late prophase, and a plus end directed motor during S-phase and G2 (Figure 7b). Such switches might involve activation or inactivation of motors of opposite polarity, or a change in one motor’s activity which subsequently influences the direction of transport via the tug-of-war motor interplay [46].

Temporal activation/inactivation of Golgi compactness in interphase is likely an important functional phenomenon, because the integrity of the Golgi complex is important for polarization of motile cells and directional cell migration [18,21,23,47]. Polarized orientation of the Golgi towards the motile cell front allows it to serve as a source of polarity factors [20] and front-directed MTs [48], which deliver post-Golgi trafficking [16], mRNA [49], and other materials to the protruding edge. Our new data indicate that Golgi configuration fine-tunes this polarity function: highly compact Golgi (C1 mode) in G1 is apparently capable of providing very focused polarity cues, while wide, equatorially extended Golgi (E mode) is radially scattered and less efficiently supports polarity. Because Golgi configuration is driven by the cell cycle, cells in G1 consistently move the fastest and most persistently (Figure 7c, left). As interphase progresses, cells slow down and cease migration when the Golgi reaches the full E mode, in time for mitosis. These data are consistent with previous observations that G2 cells migrate slower [50,51]. We observe that the Golgi-centrosome proximity is minimized at the time of slower migration speed (E mode), however it was shown that the Golgi-centrosome link is not directly needed for migration efficiency [18]. Thus, it is likely that Golgi morphology and not the centrosome link is important for cell cycle-dependent migration efficiency (Figure 7c, left).

Our research also suggests C2 mode occurs prior to NEB. The phosphorylation of nuclear envelope proteins, such as NUP [52], has been widely accepted as initiating NEB. Moreover, NEB is considered to be a concerted effort between the aforementioned mechanism and mechanical tension on the nuclear envelope [53] exerted by microtubules and resulting in the tearing of the nuclear lamina. Our data show that without the centrosomes and subsequently the centrosome microtubule array, NEB is unaffected. This suggests that the microtubules nucleated at the Golgi located in direct proximity to the nucleus (late E and C2 modes) are sufficient for providing tension for a successful NEB event. Overall, our data indicate that Golgi configuration and association with the centrosome are tightly regulated within the cell cycle. Combined with the previous knowledge, our findings indicate that each Golgi configuration is associated with a specific function and is significant for cell physiology.

## Supplementary Materials

The following are available online at https://www.mdpi.com/2073-4409/9/5/1069/s1, Figure S1: Golgi mode quantitative parameters and their correlative relationships. Figure S2: Diversity of Golgi configuration and Golgi-centrosome association in S-phase and prophase. Figure S3: Examples of radial Golgi distribution quantification. Video S1: Golgi dynamics throughout the cell cycle. Video S2: C2 mode occurs prior to NEB. Video S3: E mode progression in a control cell. Video S4: E mode progression in an acentrosomal cell. Video S5: Golgi tubule extension along the nucleus. Video S6: E mode progression in a control ice-treated cell. Video S7: E mode progression in an ice-treated cell in nocodazole. Video S8: C1 mode after a control cell division. Video S9: C1 mode after an acentrosomal cell division. Video S10: C1 mode after a division of a cell with a single centrosome. Video S11: C2 mode in a control cell. Video S12: C2 mode in an acentrosomal cell. Video S13: Centrosome movements during S/G2 transition. Video S14: Golgi and centrosome movements during the centrosome separation (1). Video S15: Golgi and centrosome movements during the centrosome separation (2). Video S16: Dynamics of radial Golgi distribution during C1 to E mode transition. Video S17: Cell migration during C1 to E mode transition (1). Video S18: Cell migration during C1 to E mode transition (2).
