# Supplementary material for: Cell Cycle-Dependent Dynamics of the Golgi-Centrosome Association in Motile Cells
Source: Cells. 2020 Apr 25;9(5):1069. doi: 10.3390/cells9051069 (PMC7290758; doi:10.3390/cells9051069)
Supplement: Supplementary file 1 [file cells-09-01069-s001.zip › Frye_Supplementary Materials/Frye_Supp Figures and Legends/Frye_Supp Figures and Legends.docx]

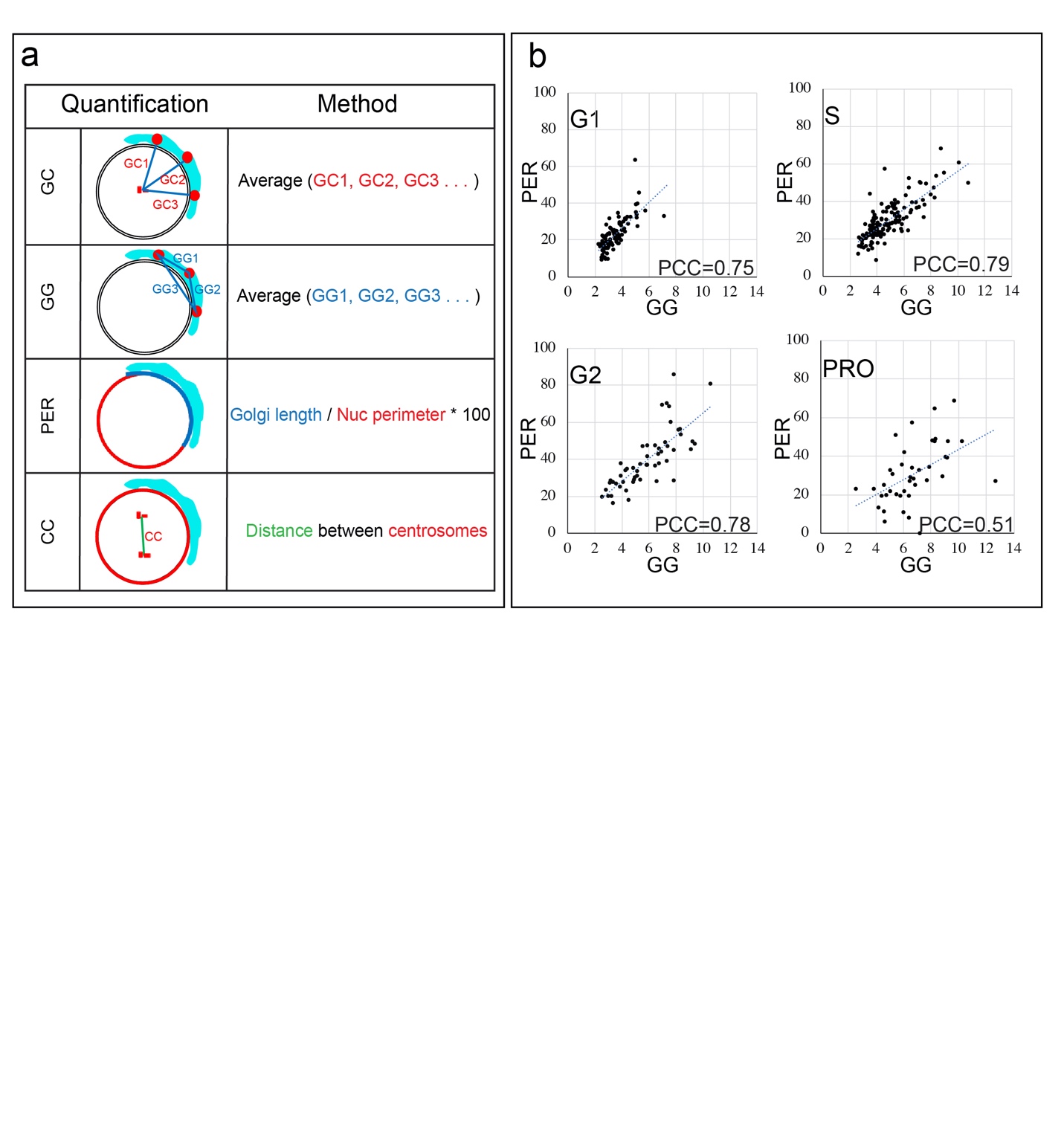


**Figure S1. Golgi mode quantitative parameters and their correlative relationships**. (**a**) Table summarizes computation of each Golgi and centrosome positioning parameter used for quantification (See Materials and Methods). (**b**) Correlation plots of 2D (PER) vs 3D (GG) Golgi configuration indices per cell cycle stage. High Pearson’s correlation coefficient (PPC) for PER vs GG correlations indicates that they can be used interchangeably to describe the degree of Golgi-centrosome association/dissociation. n=343. The data used in (**b,c**) are the same as in Figure 1b-e, based on images as in Figure 1a.


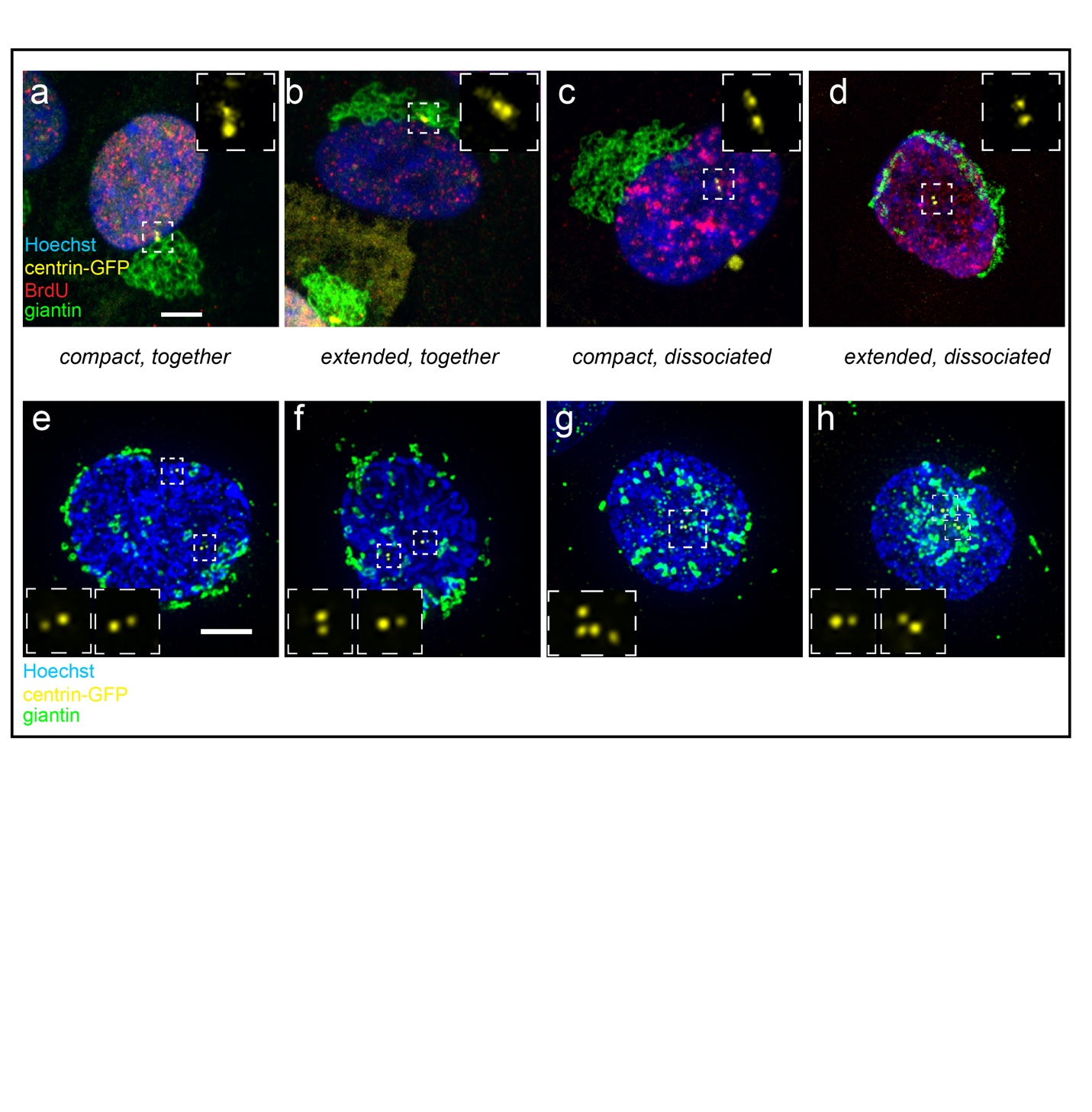


**Figure S2. Diversity of Golgi configuration and Golgi-centrosome association in S-phase and prophase**. (**a-d**) Examples of variable Golgi and centrosome positioning in S-phase. Staining: Hoechst (blue), centrin1-GFP (yellow), BrdU (red), giantin (green). Boxed regions (centrosomes) are enlarged in insets. Maximum intensity projections of laser scanning confocal stacks. Scale 5 µm. (**e-h**) Examples of variable Golgi and centrosome positioning in prophase. Staining: DAPI (blue), centrin1-GFP (yellow), giantin (green). Boxed regions (centrosomes) are enlarged in insets. Maximum intensity projections of laser scanning confocal stacks. Scale 5 µm.


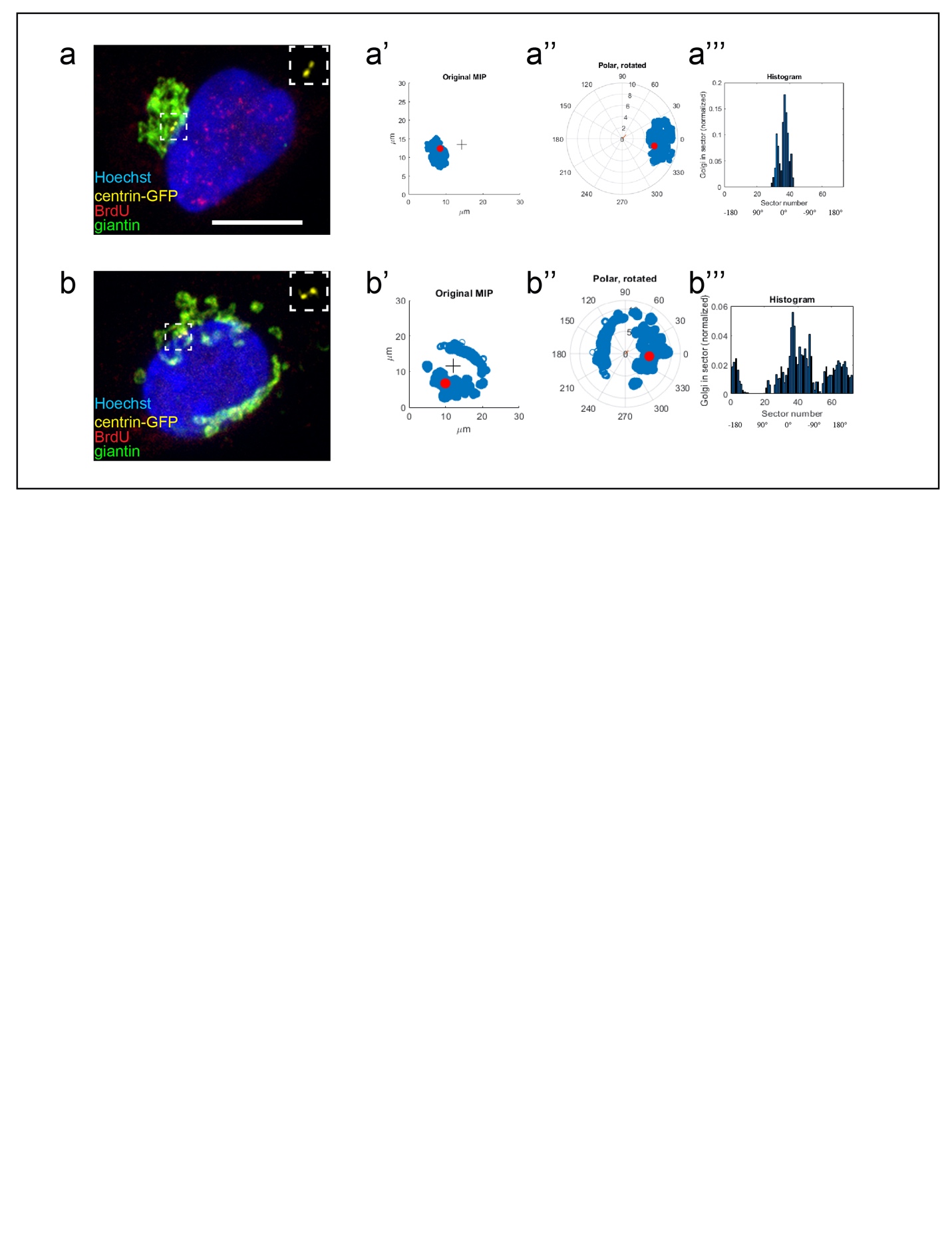
**Figure S3. Examples of radial Golgi distribution quantification**. (**a-a’**) Example of compact (high polarity) radial Golgi distribution. (b-b’) Example of equatorial (low polarity) radial Golgi distribution. (**a,b**) Maximum intensity projections of laser scanning confocal stacks. Immunostaining: Hoechst (blue), centrin1-GFP (yellow), BrdU (red), giantin (green). Scale 10 µm. (**a’, b’**) Map of Golgi density (blue) in original 2D coordinates. Centrosome center position, red. (**a’’, b’’**) Map of Golgi density (blue) in radial 2D coordinates. Centrosome center position, red. (**a’’’, b’’’**) Histograms of radial Golgi distribution for individual cells.
