# Supplementary material for: Cell Cycle-Dependent Dynamics of the Golgi-Centrosome Association in Motile Cells
Source: Cells. 2020 Apr 25;9(5):1069. doi: 10.3390/cells9051069 (PMC7290758; doi:10.3390/cells9051069)
Supplement: Supplementary file 1 [file cells-09-01069-s001.zip › Frye_Supplementary Materials/Frye_Supp Movies and Legends/Frye_Supplemental Movies Legend.docx]

**Supplemental Movies Legend**

Video S1. A time-lapse imaging sequence of an RPE1 cell stably expressing Golgi (RFP-TGN, red) and centrosome markers (centrin1-GFP, green) transitioning through the cell cycle. White arrow indicates cell position. Note transitions from C1 to E-mode, pre-mitotic C2 Golgi compaction, and post-mitotic C1 Golgi compaction. Timepoints correspond to time to mitosis (t=0 minutes) as indicated in Figure 2b-2e. Maximum intensity projection of a spinning disk confocal stack. Time, hours, minutes. Scale 10 µm. Corresponds to Figure 2a.

Video S2. A time-lapse imaging sequence of RPE1 cells transfected with GFP-giantin and mCherry-NLS. Note Golgi compaction at 41’40” and 49’20” and NLS leakage into the cytoplasm at 54’20”(corresponds to 0’ in Figure 2i). Maximum intensity projection of a spinning disk confocal stack. Time, minutes, seconds. Scale, 5 µm. Corresponds to Figure 2i.

Video S3. A time-lapse imaging sequence of RPE1 cells stably expressing Golgi (RFP-TGN, red) and centrosome markers (centrin1-GFP, green). E-mode progression within a 5 hour period in control cell after DMSO treatment for 72 hours. Maximum intensity projection of a spinning disk confocal stack. Time, hours, minutes. Scale 10 µm. Corresponds to Figure 3a.

Video S4. A time-lapse imaging sequence of RPE1 cells stably expressing Golgi (RFP-TGN, red) and centrosome markers (centrin1-GFP, green). E-mode progression within a 5 hour period pretreated with Centrinone B for 72 hours. Maximum intensity projection of a spinning disk confocal stack. Time, hours, minutes. Scale 10 µm. Corresponds to Figure 3b.

Video S5. A time-lapse imaging sequence of RPE1 cells stably expressing Golgi (RFP-TGN, red). Progressive movement of dynamic Golgi tubule (white arrows) along the nuclear equator (TGN, red) shown in 20 second intervals. Asterisk denotes starting position of Golgi membrane tubule movement. Maximum intensity projection of a spinning disk confocal stack. Time, seconds. Scale 5 µm. Corresponds to Figure 3f.

Video S6. A time-lapse imaging sequence of RPE1 cells stably expressing Golgi (RFP-TGN, red) and centrosome markers (centrin1-GFP, green). E-mode progression in cells pre-treated on ice for 45 minutes and recorded in the presence of DMSO (control). Control for Movie 7. Maximum intensity projection of a spinning disk confocal stack. Time, hours, minutes. Scale 5 µm. Corresponds Figure 3g.

Video S7. A time-lapse imaging sequence of RPE1 cells stably expressing Golgi (RFP-TGN, red) and centrosome markers (centrin1-GFP, green). E-mode is not progressing in cells pre-treated on ice for 45 minutes and recorded in the presence of nocodazole (no MTs present). Compare to control in Movie 6. Maximum intensity projection of a spinning disk confocal stack. Time, hours, minutes. Scale 5 µm. Corresponds to Figure 3h.

Video S8. A time-lapse imaging sequence of RPE1 cells stably expressing Golgi (RFP-TGN, red) and centrosome markers (centrin1-GFP, green). C1 mode progression in control cells after DMSO treatment for 72 hours. White arrows indicate daughter cells with centrosomes. Maximum intensity projection of entire spinning disk confocal stacks. Time, hours, minutes. Scale 20 µm. Corresponds to Figure 4a.

Video S9. A time-lapse imaging sequence of RPE1 cells stably expressing Golgi (RFP-TGN, red) and centrosome markers (centrin1-GFP, green). C1-mode progression within a 5 hour period pretreated with Centrinone B for 72 hours. Yellow arrows indicate daughter cells with no centrosomes. Maximum intensity projection of entire spinning disk confocal stacks. Time, hours, minutes. Scale 10µm. Corresponds to Figure 4b.

Video S10. A time-lapse imaging sequence of RPE1 cells stably expressing Golgi (RFP-TGN, red) and centrosome markers (centrin1-GFP, green). C1-mode progression within a 5 hour period pretreated with Centrinone B for 72 hours. White arrows indicate daughter cells with centrosomes. Yellow arrows indicate daughter cells with no centrosome. Maximum intensity projection of entire spinning disk confocal stacks. Time, hours, minutes. Scale 10 µm. Corresponds to Figure 4c.

Video S11. A time-lapse imaging sequence of RPE1 cells stably expressing Golgi (RFP-TGN, red) and centrosome markers (centrin1-GFP, cyan). C2 mode progression in control cells after DMSO treatment for 72 hours. Maximum intensity projection of ventral (8.0 µm) or dorsal (8.0 µm) segments of the cell. Time, hours, minutes. Scale 10 µm. Corresponds to Figure 4f.

Video S12. A time-lapse imaging sequence of RPE1 cells stably expressing Golgi (RFP-TGN, red) and centrosome markers (centrin1-GFP, cyan). C2 mode progression cells pretreated with Centrinone B for 72 hours. Maximum intensity projection of ventral (4.0 µm) or dorsal (4.0 µm) segments of the cell. Time, hours, minutes. Scale 10 µm. Corresponds to Figure 4g.

Video S13. Time-lapse multi-mode DIC (left) and wide-field epifluorescence (right) 3D time lapse recordings of RPE1 cells stably expressing GFP-centrin (greyscale) during S/G2 transition. The duplicated centrosome resides within a common complex on the ventral surface of the nucleus at -1h25’ to -1h00’. One hour prior to mitosis, the centrosomes begin to separate and reach edges of the nucleus ~ 30’ prior to NEB. Then one centrosome migrates to the center of the dorsal nuclear surface while the second centrosome returns to the center of the ventral nuclear surface. Concurrently with NEB (00h00’), centrosomes begin to separate steadily to form mitotic spindle poles. Centrin-GFP fluorescence frames are maximum intensity projections (top right, XY view; bottom right, XZ view). DIC frames are single focal planes. Time to NEB shown in hours, minutes. Scale 10 µm. Corresponds to Figure 5a.

Video S14. A time-lapse imaging sequence of RPE1 cells stably expressing Golgi (RFP-TGN, red) and centrosome markers (centrin1-GFP, green) during centrosome separation overtime. White arrows indicate centrosome positions. Maximum intensity projection of entire spinning disk confocal stacks. Time, hours, minutes. Scale 10 µm. Timepoints correspond to maximal CC distance (t=0mins) as shown in Figure 5c,d.

Video S15. A time-lapse imaging sequence of RPE1 cells stably expressing Golgi (RFP-TGN, red) and centrosome markers (centrin1-GFP, green) during centrosome separation overtime. White arrow indicates cell position. Maximum intensity projection of entire spinning disk confocal stacks. Time, hours, minutes. Scale 10 µm. (additional example of Golgi-centrosome dissociation overtime).

Video S16. A time-lapse imaging sequence of RPE1 cells stably expressing Golgi (RFP-TGN, red) and centrosome markers (centrin1-GFP, green). White arrows indicate cell position. Inset is histograms of radial Golgi distribution in the cell, corresponding to each timepoint. Maximum intensity projection of entire spinning disk confocal stacks. Time, hours, minutes. Scale 5 µm. Corresponds to Figure 6a, 5d’.

Video S17. A time-lapse epi-fluorescence imaging sequences of RPE1 cells stably expressing Golgi marker (RFPTGN). Images are pseudo-color-coded according to Golgi configuration modes: compact Golgi (PER <25%, red), early E-mode (PER=25-50%, yellow), and advanced E-mode (PER>50%, green). Frames with 10 minute intervals are overlaid for all timepoints. Time, hours, minutes. Scale, 20 µm. Corresponds to Figure 6d.

Video S18. A time-lapse epi-fluorescence imaging sequences of RPE1 cells stably expressing Golgi marker (RFPTGN). Images are pseudo-color-coded according to Golgi configuration modes: compact Golgi (PER <25%, red), early E-mode (PER=25-50%, yellow), and advanced E-mode (PER>50%, green). Frames with 10 minute intervals are overlaid for all timepoints. Time, hours, minutes. Scale, 20 µm. Corresponds to Figure 6e.
