# Supplementary figures and images for: Cell Cycle-Dependent Dynamics of the Golgi-Centrosome Association in Motile Cells
Source: Cells. 2020 Apr 25;9(5):1069. doi: 10.3390/cells9051069 (PMC7290758; doi:10.3390/cells9051069)

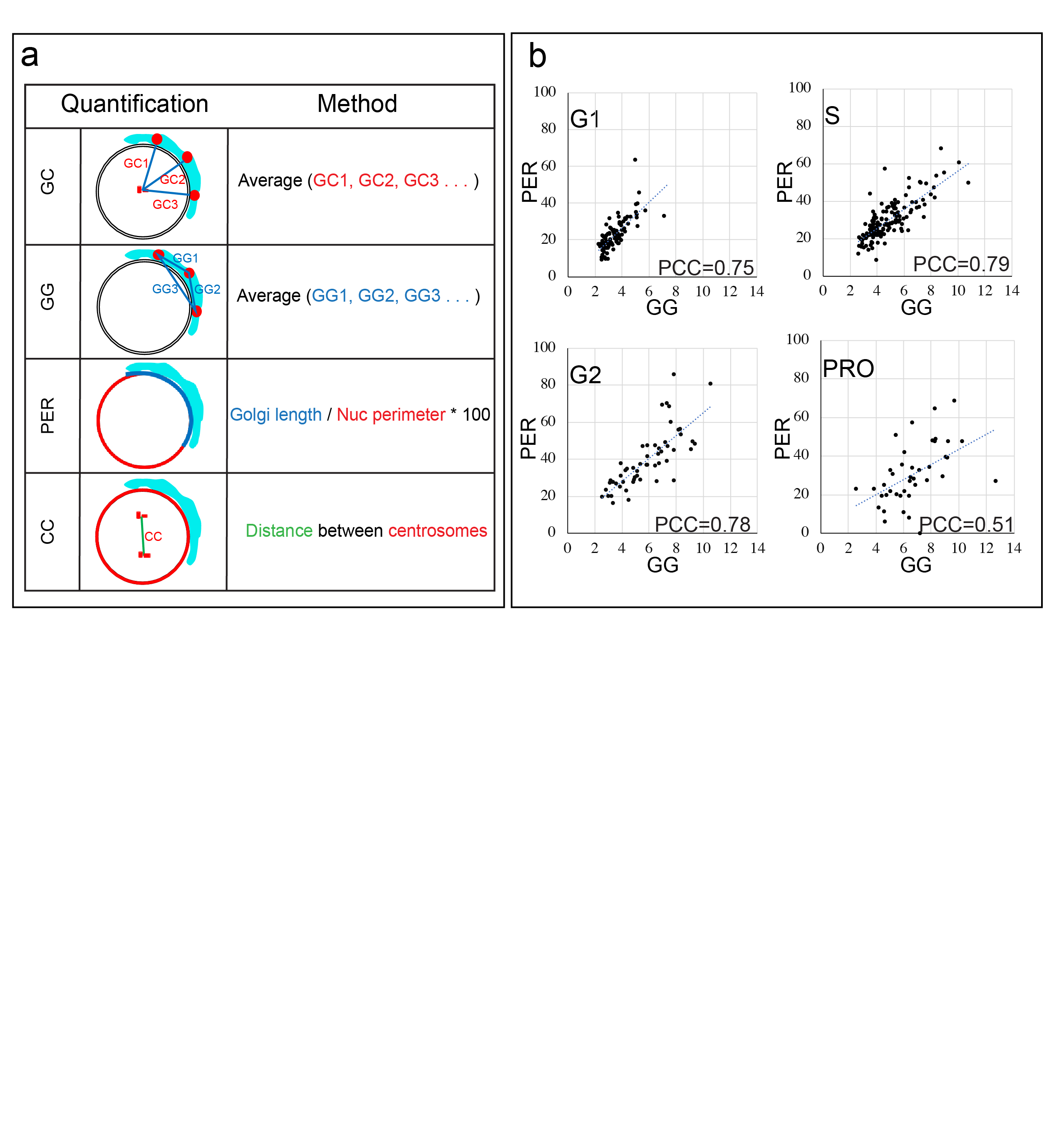

Supplement: Supplementary file 1 [file cells-09-01069-s001.zip › Frye_Supplementary Materials/Frye_Supp Figures and Legends/Supp Figure 1.jpg]

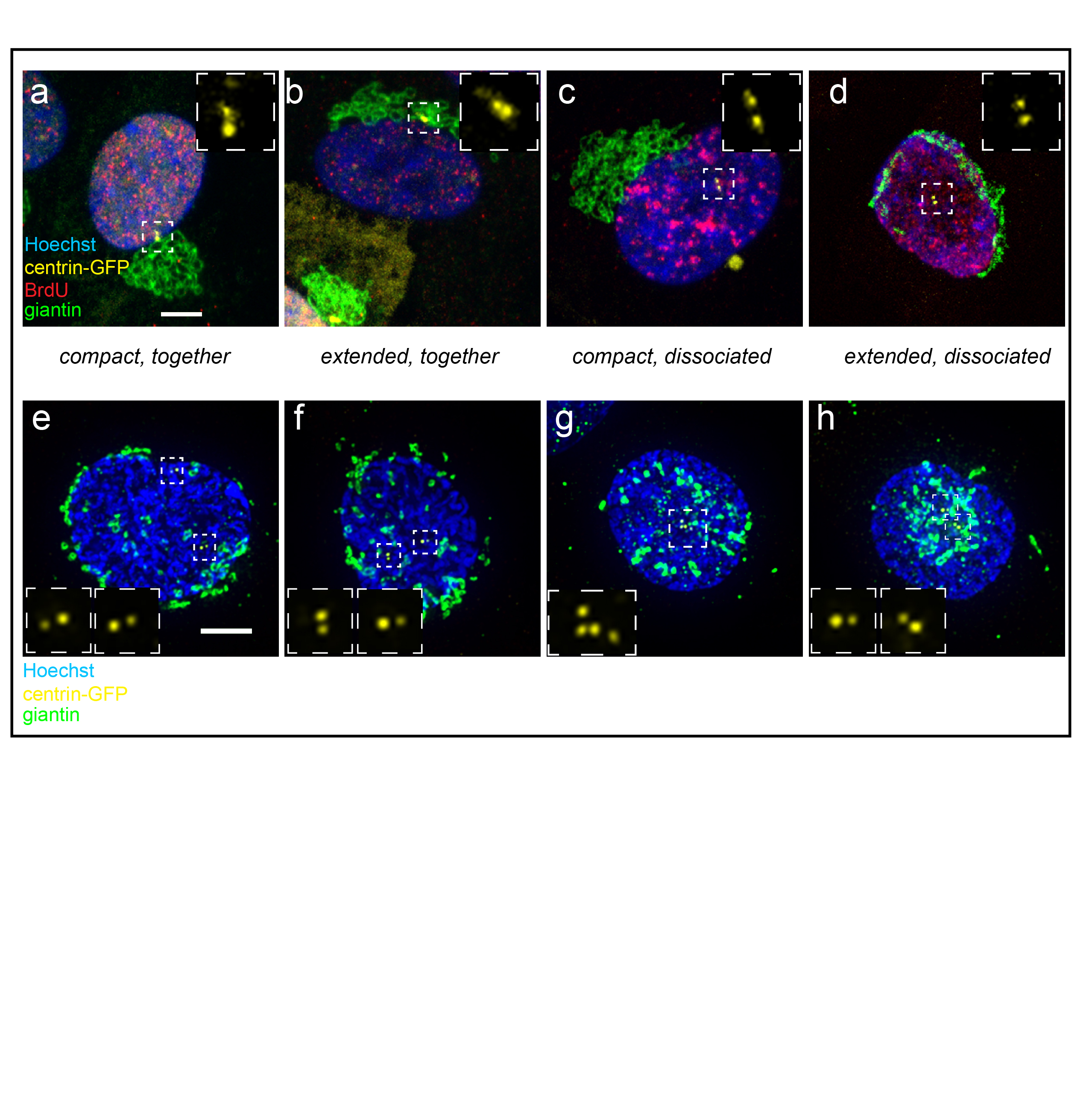

Supplement: Supplementary file 1 [file cells-09-01069-s001.zip › Frye_Supplementary Materials/Frye_Supp Figures and Legends/Supp Figure 2.jpg]

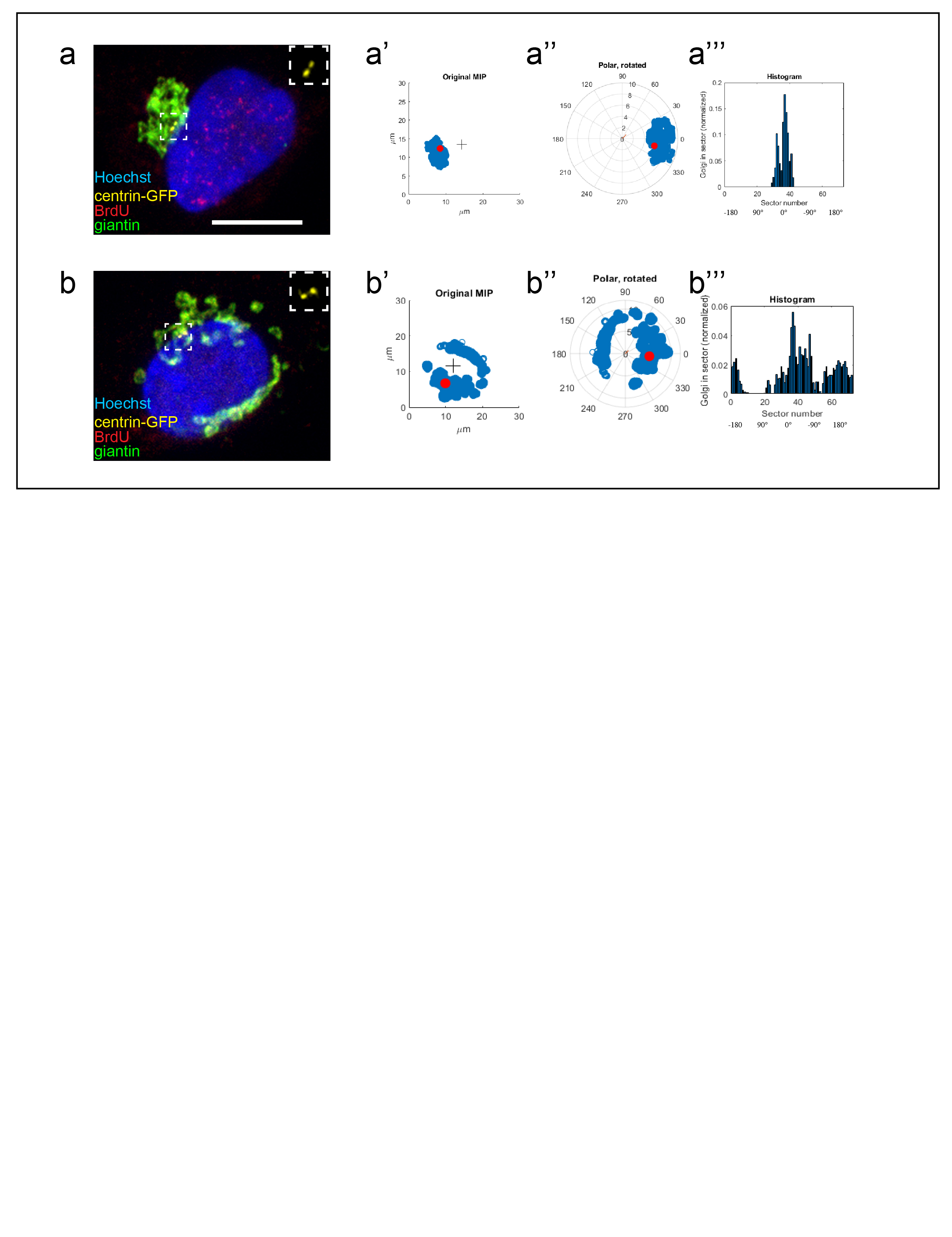

Supplement: Supplementary file 1 [file cells-09-01069-s001.zip › Frye_Supplementary Materials/Frye_Supp Figures and Legends/Supp Figure 3.jpg]
